# PICO Questions and DELPHI Methodology for the Management of Venous Thromboembolism Associated with COVID-19

**DOI:** 10.3390/v13112128

**Published:** 2021-10-22

**Authors:** Antoni Riera-Mestre, Luis Jara-Palomares, Ramón Lecumberri, Javier Trujillo-Santos, Enric Grau, Angeles Blanco-Molina, Ana Piera Carbonell, Sonia Jiménez, Manuel Frías Vargas, Mari Paz Fuset, Sergi Bellmunt-Montoya, Manuel Monreal, David Jiménez, on behalf of the COVILAX Project

**Affiliations:** 1 Internal Medicine Department, Hospital Universitari de Bellvitge, Bellvitge Biomedical Research Institute (IDIBELL), 08907 Barcelona, Spain; 2 Faculty of Medicine and Health Sciences, Universitat de Barcelona, 08907 Barcelona, Spain; 3 Medical-Surgical Unit for Respiratory Diseases, Virgen del Rocío University Hospital, 28029 Sevilla, Spain; luisoneumo@hotmail.com; 4 Center for Biomedical Research in Respiratory Diseases Network (CIBERES), Instituto de Salud Carlos III, 28029 Madrid, Spain; djimenez.hrc@gmail.com; 5 Hematology Department, Clínica Universidad de Navarra, 31008 Pamplona, Spain; rlecumber@unav.es; 6 Center for Biomedical Research Network on Cardiovascular Diseases (CIBERCV), Instituto de Salud Carlos III, 28029 Madrid, Spain; 7 Internal Medicine Department, Hospital General Universitario Santa Lucía, 30204 Cartagena, Spain; javier.trujillosantos@gmail.com; 8 Faculty of Health Sciences, Universidad Católica San Antonio de Murcia (UCAM), 30107 Murcia, Spain; mmonreal.germanstrias@gencat.cat; 9 Hematology Department, Lluis Alcanyis de Xativa Hospital, 46800 Valencia, Spain; grau_enr@gva.es; 10 Internal Medicine Unit, Hospital Universitario Reina Sofía, 10004 Córdoba, Spain; mablancom@telefonica.net; 11 Luanco Health Center, 33440 Gozón, Spain; apiecar@gmail.com; 12 Emergency Area, Hospital Clínic, Institut d’Investigacions Biomèdiques August Pi i Sunyer (IDIBAPS), 08036 Barcelona, Spain; sjimenez131707@gmail.com; 13 Comillas Health Center, 28019 Madrid, Spain; manueldejesus.frias@salud.madrid.org; 14 Intensive Medicine Department, Hospital Universitari de Bellvitge, Bellvitge Biomedical Research Institute (IDIBELL), 08907 Barcelona, Spain; mfuset@bellvitgehospital.cat; 15 Angiology and Vascular Surgery Department, Hospital Universitari Vall d’Hebron, 08035 Barcelona, Spain; sbellmunt@vhebron.net; 16 Surgery Department, Universitat Autònoma de Barcelona, 08193 Barcelona, Spain; 17 Vall d’Hebron Institut de Recerca, 08035 Barcelona, Spain; 18 Internal Medicine Department, Germans Trias i Pujol University Hospital, 08916 Badalona, Spain; 19 Pulmonology Department, Ramón y Cajal Hospital (IRYCIS), 28034 Madrid, Spain; 20 Department of Medicine, Alcala University, 28805 Madrid, Spain

**Keywords:** venous thromboembolic disease, COVID-19, coronavirus, pulmonary embolism, deep vein thrombosis, anticoagulation

## Abstract

Patients with coronavirus disease 2019 (COVID-19) have a higher risk of venous thromboembolic disease (VTE) than patients with other infectious or inflammatory diseases, both as macrothrombosis (pulmonar embolism and deep vein thrombosis) or microthrombosis. However, the use of anticoagulation in this scenario remains controversial. This is a project that used DELPHI methodology to answer PICO questions related to anticoagulation in patients with COVID-19. The objective was to reach a consensus among multidisciplinary VTE experts providing answers to those PICO questions. Seven PICO questions regarding patients with COVID-19 responded with a broad consensus: 1. It is recommended to avoid pharmacological thromboprophylaxis in most COVID-19 patients not requiring hospital admission; 2. In most hospitalized patients for COVID-19 who are receiving oral anticoagulants before admission, it is recommended to replace them by low molecular weight heparin (LMWH) at therapeutic doses; 3. Thromboprophylaxis with LMWH at standard doses is suggested for COVID-19 patients admitted to a conventional hospital ward; 4. Standard-doses thromboprophylaxis with LMWH is recommended for COVID-19 patients requiring admission to Intensive Care Unit; 5. It is recommended not to determine D-Dimer levels routinely in COVID-19 hospitalized patients to select those in whom VTE should be suspected, or as a part of the diagnostic algorithm to rule out or confirm a VTE event; 6. It is recommended to discontinue pharmacological thromboprophylaxis at discharge in most patients hospitalized for COVID-19; 7. It is recommended to withdraw anticoagulant treatment after 3 months in most patients with a VTE event associated with COVID-19. The combination of PICO questions and DELPHI methodology provides a consensus on different recommendations for anticoagulation management in patients with COVID-19.

## 1. Introduction

Coronavirus disease 2019 (COVID-19) mainly affects the respiratory system but is frequently accompanied by extrapulmonary manifestations [1,2]. There is strong evidence that patients with COVID-19 have a higher risk of arterial thrombosis and venous thromboembolic disease (VTE) than patients with other infectious or inflammatory diseases [3,4,5]. This increased risk of VTE, both in the form of deep vein thrombosis (DVT) and pulmonary thromboembolism (PTE), is due to the presence of hypercoagulability, endothelial damage, and venous stasis [2,3,5]. In addition, in some patients with COVID-19, pulmonary inflammation and local endothelial damage with subsequent complement system activation, thrombin generation, platelet and leukocyte recruitment, and the initiation of innate and adaptive immune responses, may lead to in situ pulmonary microthrombosis (thromboinflammation) [3,6].

Although there are numerous publications on the prevention and treatment of VTE in patients with COVID-19, the evidence generated to date has not been sufficiently consistent to make solid recommendations [7,8,9,10]. For this reason, the aim of the present study was to seek a consensus among VTE experts on the use of anticoagulation in patients with COVID-19.

## 2. Materials and Methods

The COVILAX project (COVID-19 and thromboprophyLAXis of VTE) is a multidisciplinary consensus among experts in the management of patients with VTE. The aim was to improve knowledge of COVID-19-associated VTE and to provide clinicians with consensus recommendations in those areas that are more controversial and have less scientific evidence in prevention, diagnosis, and treatment. The study was approved by the Clinical Research Ethics Committee of the Hospital Universitari de Bellvitge (Barcelona, Spain; ethic approval number PR361/21). 

The duration of the project was 6 months, between March and August 2021. The main objective was to reach a consensus on thromboprophylaxis, diagnosis, and treatment of VTE in patients with COVID-19 in different clinical situations, especially regarding the use of anticoagulant drugs. The methodology used established a set of clinical issues using PICO (Patient, Intervention, Comparison, Outcomes) questions and then applied DELPHI method to provide responses [11,12]. DELPHI is a structured methodology that systematically collects expert opinions on a particular issue and builds a general group consensus [13]. The benefits of the DELPHI methodology have been widely described in the literature [11,12,13]. A panel of 13 national experts was recruited for the project, which was developed in four phases. In phase 1, a group of experts (ARM, RL, SJ, MFV, MM, and DJ) defined the domains and PICO questions to be addressed. In phase 2, another group of experts (LJP, JTS, EG, ABM, APC, MPF, and SBM) discussed and answered the PICO questions. In phase 3, the first group discussed the answers elaborated by the second group and after several rounds of feedback, drafted a response for each PICO question. In phase 4, each member of the panel individually expressed his/her agreement or disagreement with the answers to the PICO questions, and the degree of agreement was recorded. Throughout the project there was an active discussion and continuous feedback via the WITHIN3 platform (https://www.within3.com/), with individual password access.

The panel used the terms “recommended” or “suggested”, if the strength of the recommendation was strong or weak, respectively [14]. The term standard-doses of pharmacological thromboprophylaxis refers to the usual doses of the different low-molecular-weight heparins (LMWH) for thromboprophylaxis according to their approved drug labeling, to differentiate them from intermediate or therapeutic anticoagulant doses.

## 3. Results

Four domains and seven PICO questions were defined (Table 1). Overall, there was an initial consensus on two general recommendations that affect all PICO questions. Firstly, a dose adjustment of LMWH is recommended in obese patients and in patients with renal insufficiency. In the case of patients with obesity (defined by a body mass index > 30 kg/m^2^ or weight > 100 kg), the adjustment would be made according to different recommendations [15,16,17]. In patients with renal failure (defined by a glomerular filtration rate < 30 mL/min), the recommended dose in each medication data sheet of the different LMWHs should be used [18]. Secondly, it is recommended to consider risk factors for bleeding (Table 2) and to assess bleeding risk individually in all patients with COVID-19 before initiating any anticoagulant therapy, although no agreement was reached on how this risk should be graded.

### 3.1. Domain 1: Thromboprophylaxis in COVID-19 Patients Not Requiring Hospital Admission

#### PICO 1: Which Patients with COVID-19 Not Requiring Hospital Admission Should Receive Standard-Doses of Pharmacological Thromboprophylaxis?

1. It is recommended to avoid pharmacological thromboprophylaxis in most COVID-19 patients that do not require hospital admission.

2. It is suggested that standard-doses of thromboprophylaxis might be administered to those patients with symptomatic COVID-19 and risk factors for VTE (Table 3), although not requiring hospital admission.

Consensus: 13/13

Comments: There are no strong data to support routine use of standard-doses of pharmacological thromboprophylaxis in these patients. A randomized clinical trial (RCT) was published evaluating the efficacy and safety of sulodexide in patients with COVID-19 who did not require hospital admission [19]. In this RCT, sulodexide significantly reduced the need for hospital admission compared to placebo (17.7% versus 29.4%) at 21 days. Some major limitations of this trial are the lack of double-blinding, the small sample size, and the heterogeneity of the concomitant treatments received by both groups. Pending the results of ongoing RCTs (ClinicalTrials.gov: NCT04400799, NCT04492254), the panel considered the increased risk of VTE in COVID-19 patients who have additional thrombotic risk factors (Table 3), even if not requiring hospital admission, and suggests pharmacological thromboprophylaxis when any of these factors coexist [2,20].

### 3.2. Domain 2: Thromboprophylaxis in Hospitalized COVID-19 Patients

#### 3.2.1. PICO 2: In Hospitalized Patients for COVID-19 Who Were Receiving Anticoagulation with Oral Anticoagulants Prior to Admission, Should We Substitute Them with LMWH?

1. In most hospitalized patients for COVID-19 who were receiving oral anticoagulants before admission, it is recommended to replace them by LMWH at therapeutic doses.

2. It is suggested to maintain treatment with vitamin K antagonists (VKA) in patients with mechanical heart valves who require hospitalization for COVID-19, if clinically stable.

Consensus: 13/13

Comments: Patients with COVID-19 who require hospital admission receive treatments that could modify the effect of oral anticoagulants, mainly VKA [21]. In addition, these patients often require invasive procedures and may have difficulties with oral tolerance [22,23]. The use of LMWH during hospital admission minimizes the risk of drug-drug interactions and facilitates the performance of invasive procedures [7,8]. However, the use of VKAs is the recommended treatment for patients with mechanical heart valves. There are no data to support the use of non-vitamin K oral anticoagulants in these patients. The interruption of VKAs for planned invasive procedures or hospital admission for other reasons, such as COVID-19 infection, is a complex issue associated with multiple factors including the type and location of the prosthetic valve, reason for interruption, thromboembolic risk, and the duration of interruption. For clinically unstable patients, therapeutic bridging with either UFH or LMWH is required, although the evidence is scarce and a case-by-case evaluation should be performed [24].

#### 3.2.2. PICO 3: What Is the Optimal Dose of LMWH for Most COVID-19 Patients Admitted to a Conventional Hospital Ward?

1. Thromboprophylaxis with LMWH at standard doses (versus therapeutic anticoagulation) is suggested for COVID-19 patients admitted to a conventional hospital ward, unless contraindicated.

Consensus: 13/13

Comments: The main objectives of the different RCTs that analyze the possible benefits of therapeutic anticoagulation are not focused on VTE prevention but on COVID-19 treatment [22,25]. In the ACTION RCT, which enrolled stable (more than 93%) and unstable hospitalized patients with COVID-19, therapeutic anticoagulation with rivaroxaban 20 mg/day (in clinically stable patients) or enoxaparin 1 mg/kg/12 h (in clinically unstable patients) was not superior to standard doses of thromboprophylaxis with enoxaparin in terms of the hierarchical composite endpoint of mortality, hospital stay, or days free from requiring oxygen therapy [22]. A recent multi-platform RCT (mpRCT) compared therapeutic-dose anticoagulation with LMWH with standard-dose thromboprophylaxis with LMWH in non-critically patients with COVID-19. The results showed an adjusted absolute difference in organ support, with free days up to day 21 among patients who survived to hospital discharge of 4% in favor of therapeutic-dose anticoagulation (95% credible interval, 0.5–7.2) [25]. During hospitalization, 16 (1.4%) venous thrombotic events occurred in patients receiving therapeutic anticoagulation and 26 (2.5%) in those receiving standard-dose thromboprophylaxis. A major bleeding episode occurred in 1.9% (22/1180) of patients receiving therapeutic anticoagulation (three of them fatal), and in 0.9% (9/1047) of those receiving standard-dose thromboprophylaxis (one of them fatal). The open-label design, the different definitions used for critical and non-critical patients in the three platforms, the lack of detailed screening data to compare the most common reasons for exclusion from the trial, and the potential for ascertainment bias of major bleeding episodes are the main limitations of this study. Preliminary results of the RAPID RCT did not demonstrate a benefit of therapeutic anticoagulation with heparin compared to standard-dose thromboprophylaxis in patients admitted to a conventional hospital ward for COVID-19 and elevated D-dimer in terms of the combined primary endpoint of ICU admission or mechanical ventilation (invasive or non-invasive) or death at 28 days (16.2% vs. 21.9%). In the same RCT, the group receiving therapeutic anticoagulation had lower mortality than the group receiving standard-dose thromboprophylaxis (1.8% vs. 7.6%). Surprisingly, the risk of bleeding was lower, although not statistically significant, in the group that received therapeutic anticoagulation than in the group that received standard doses of thromboprophylaxis (0.9% vs. 1.7%) [26]. However, this RCT resulted as underpowered because at 75% of the originally planned sample size an interim analyses was pre-specified and established that the sample should be increased if the conditional power was between 60 and 80%. Although the conditional power obtained was below 60%, the sample size was not increased, thus RAPID trial remained underpowered for the analysis of the primary outcome. Moreover, when the authors performed a meta-analysis combining the results of the mpRCT in non-critically ill patients, the statistically significant reduction in overall mortality disappeared (6.4% vs. 8.1%) and the risk of bleeding was higher in those who received therapeutic anticoagulation, although not statistically significant (1.7% vs. 1.0%) [25,26]. The HEP-COVID is a multicenter, pragmatic, pseudo-blinded RCT to evaluate efficacy and safety of therapeutic anticoagulant doses compared with intermediate/standard-doses thromboprophylaxis of LMWH in hospitalized COVID-19 patients with requirement for supplemental oxygen and a D-dimer > 4× upper limit of normal or sepsis-induced coagulopathy (SIC) score ≥ 4. The primary composite endpoint was VTE, arterial thromboembolic events, and all-cause mortality at 30 days. Preliminary results in non-ICU patients randomized to therapeutic LMWH doses (*n* = 84) showed a statistically significant reduction of the primary endpoint compared to intermediate/standard doses of thromboprophylaxis of LMWH (*n* = 86) (16.7% vs. 36.1%). The risks of major bleeding in these non-ICU patients were similar (2.38% vs. 2.33%) [27].

#### 3.2.3. PICO 4: What Is the Optimal Dose of LMWH for Most COVID-19 Patients Admitted to an Intensive Care Unit (ICU)?

1. Standard-doses thromboprophylaxis with LMWH (versus intermediate doses or therapeutic anticoagulation) is recommended for COVID-19 patients requiring admission to an ICU, unless there is contraindication.

2. A high suspicion index of VTE is recommended in COVID-19 patients admitted to ICU, particularly when there is clinical worsening with no evident alternative diagnosis.

Consensus: 12/13

Comments: In the INSPIRATION RCT, patients admitted to ICU for COVID-19 were openly randomized to receive intermediate-dose enoxaparin (1 mg/kg/d) or standard-dose thromboprophylaxis (40 mg/d). Intermediate dose administration did not significantly reduce the primary efficacy event, which was a combination of arterial or venous thrombosis, need for extracorporeal membrane oxygenation (ECMO), or 30-day mortality (45.7% vs. 44.1%), and caused more major bleeding episodes (2.5% vs. 1.4%) [23]. The mpRCT for critically ill patients with COVID-19 was stopped early due to futility in the primary outcome of organ support, with free days up to day 21 among patients who survived to hospital discharge. Moreover, the results showed no statistically significant differences between therapeutic anticoagulation or standard-dose thromboprophylaxis in survival (62.7% vs. 64.5%) and there were more major bleeding events in patients that received therapeutic anticoagulation (3.8% vs. 2.3%) [28]. Regarding the use of intermediate doses, a recent RCT compared the use of standard-doses thromboprophylaxis with intermediate doses of enoxaparin in patients with COVID-19 admitted to ICU and/or had laboratory evidence of coagulopathy, defined by a modified International Society on Thrombosis and Haemostasis (ISTH) with an overt disseminated intravascular coagulation (DIC) score ≥3. This RCT also found no significant differences in the primary endpoint of 30-day overall mortality (21% vs. 15%) [29]. Preliminary results of the HEP-COVID RCT in ICU patients showed no benefit in the primary composite endpoint (VTE, arterial thromboembolic events and all-cause mortality at 30 days) for those randomized to therapeutic LMWH doses (*n* = 45) compared to intermediate/standard-doses thromboprophylaxis of LMWH (*n* = 38) (51.1% vs. 55.3%) and higher risk of bleeding (8.9% vs. 0%) [27].

In critically ill patients with COVID-19, it is not always possible to perform the usual diagnostic imaging tests (chest CT angiography, compression echography of the LMII, echocardiography), or the results are inconclusive when VTE is suspected. In this scenario, anticoagulation could be considered without diagnostic confirmation, once the patient’s bleeding risk assessed is low [30].

### 3.3. Domain 3: Diagnosis and Treatment Guide of VTE in COVID-19 Hospitalized Patients

#### PICO 5: Which Is the Significance of High D-Dimer Levels in Hospitalized Patients for COVID-19?

1. It is recommended not to determine D-Dimer levels routinely in COVID-19 hospitalized patients as a strategy to select those in whom VTE should be suspected, or as a part of the diagnostic algorithm for clinical suspicion to rule out or confirm a VTE event.

2. It is recommended not to use D-dimer levels to select the intensity of thromboprophylaxis with LMWH in patients requiring admission for COVID-19.

Consensus: 13/13

Comments: In patients with COVID-19, elevated D-dimer levels are the result of the patient’s inflammatory state and hypercoagulability [2,31]. High D-dimer levels have been associated with more severe forms of the disease and with a higher risk of bleeding, VTE, and mortality [2,30,32,33,34,35]. However, in these cases, D-dimer is not a screening test for VTE (to identify those in whom VTE should be suspected). In addition, most patients with COVID-19 have elevated D-dimer levels, therefore, its usefulness in ruling out VTE associated with COVID-19 without the need for imaging tests is reduced [2,4,33,34]. Moreover, there is no evidence that isolated levels of D-dimer justify a change in antithrombotic therapy. In fact, the effect of anticoagulation in both critically and noncritically ill patients, according to the results of the corresponding mpRCT, was not influenced by stratification according to D-dimer levels [25,28]. Although cut-off levels with better sensitivity and specificity were described in a retrospective series of patients, a unanimous diagnostic cut-off point for VTE has not been defined [2,31,32,33,34,35]. However, VTE suspicion should arise in patients with clinical deterioration associated with high D-dimer levels and no elevation of other inflammatory markers.

### 3.4. Domain 4: Follow-Up after Discharge of Patients Admitted for COVID-19 with and without a Vte Event

#### 3.4.1. PICO 6. Which Patients without COVID-19-Associated VTE Should Receive Extended Standard-Doses Thromboprophylaxis after Hospital Discharge?

1. It is recommended to discontinue pharmacological thromboprophylaxis at discharge in most patients hospitalized for COVID-19, after a minimum of 6 days.

2. Extended duration of thromboprophylaxis with standard-doses of LMWH is suggested after hospital discharge to those patients with risk factors for VTE (Table 3).

Consensus: 12/13

Comments: There are no strong data to support the use of extended thromboprophylaxis after hospital discharge in these patients. However, it is well known that the risk of VTE and mortality from PE remains high in the period following discharge from acute medical illness [7,8]. The use of extended thromboprophylaxis in medical patients was evaluated in five RCTs. One compared rivaroxaban 10 mg/day versus placebo and the other four compared extended regimens (up to 42 days) of enoxaparin 40 mg/day, apixaban 2.5 mg/12 h, rivaroxaban 10 mg/day, and betrixaban 80 mg/day, versus enoxaparin 40 mg/day for a minimum of 6–14 days [36]. A meta-analysis including these five RCTs concluded that extended thromboprophylaxis reduces symptomatic VTE but increases the risk of bleeding [36]. While some RCT results are pending (ClinicalTrials.gov: NCT04650087l, NCT04662684), the panel took into account the increased risk of VTE in patients with COVID-19 who present other additional thrombotic risk factors (Table 3) and suggest extending thromboprophylaxis at discharge when any of these factors coexist [2,20,36].

#### 3.4.2. PICO 7. Which Is the Optimal Duration of Anticoagulant Treatment for Patients with COVID-19-Associated VTE?

1. It is recommended to withdraw anticoagulant treatment after 3 months in most patients with a VTE event associated with COVID-19.

2. It is suggested that the extension of anticoagulant treatment beyond the first 3 months should be evaluated in patients with respiratory symptoms associated with persistent COVID-19, particularly if there is the presence of persistently elevated inflammatory markers.

Consensus: 13/13

Comments: The risk of recurrent VTE after anticoagulant therapy withdrawal is low in patients with a transient major risk factor, as is the case of COVID-19 hospitalization [7,8]. For this reason, a duration of anticoagulant treatment of 3 months is recommended for these patients [7,8]. Durations of less than 3 months have been associated with an increased risk of VTE recurrence, and longer durations have been associated with an increased risk of bleeding [7,8,37]. As in other clinical situations, it is recommended to extend anticoagulant treatment if the triggering risk factor is maintained [7,8]. Therefore, extended anticoagulation beyond 3 months could be considered for those patients with COVID-19-associated VTE and respiratory symptoms related to persistent COVID-19 during follow-up, particularly in the presence of elevated inflammatory markers [1,2,31].

## 4. Discussion

The pathophysiology of VTE development associated with COVID-19 infection is currently being unraveled. There is strong evidence that endothelial damage generated by SARS-CoV-2 Coronavirus (Severe Acute Respiratory Syndrome Coronavirus-2) is one of the main reasons for the excess of thrombotic events associated with COVID-19, compared to other infectious or inflammatory diseases. The direct effects of SARS-CoV-2 on pneumocytes and endothelium and the interaction between the systemic inflammatory response with the hemostatic system have been deemed the linchpin of the hypercoagulable state in COVID-19. In less than 20% of infected patients, a poorly controlled viral replication leads to apoptosis of pneumocytes and endothelial cells, which activate platelets and induce coagulation factors, increase cytokines production, and activate the complement system. The so-called cytokine storm fuels these proinflammatory and procoagulatory responses further, resulting in systemic endotheliitis, capillary leakage, endothelial and organ dysfunction, and overt activation of the coagulation cascade that leads to a high incidence of microthrombi and macrothrombi and the subsequent need for organ support [1,2,3,4]. This thrombotic state is the result of a process known as immunothrombosis, in which the immune and coagulation systems cooperate to block pathogens, such as SARS-CoV-2, and limit their spread. In COVID-19 infection, a deregulated and exaggerated process of immunothrombosis, mainly involving the pulmonary microcirculation, drives the severe clinical manifestations of COVID-19 [38].

However, how to control the hypercoagulable state generated by this endothelial dysfunction remains controversial. Regarding the use of anticoagulation with heparin at therapeutic doses, all the RCTs carried out in critically ill patients have obtained similar results, with no evidence of clinical benefit [23,27,28,29]. Therapeutic anticoagulation may be unable to influence the inflammatory cascade and extensive pulmonary microthrombosis secondary to severe endothelial damage, which is characteristic of these critically ill patients [2,3].

It remains to be confirmed whether early administration of therapeutic anticoagulation in non-critically ill patients prevents progression to this extensive endothelial damage and pulmonary microthrombosis and improves prognosis. In addition to an optimal route of administration for the COVID-19 patient profile, the pleiotropic (anti-inflammatory or antiviral) effects of LMWH may provide additional benefits over oral anticoagulants [22,39]. These pleiotropic effects could have influenced the benefits obtained with higher doses of LMWH obtained in the non-critically ill patient mpRCT, where only approximately 60% of patients received corticotherapy [25]. In addition, the limitations inherent to the design of the mpRCTs should be highlighted, where the control group is not concurrent with the experimental group and the patients enter and exit the trial at different times [40]. It should also be considered that in the non-critically ill patient mpRCT, 20.4% of the anticoagulation group did not receive therapeutic doses and 26.5% of the control group received intermediate doses of LMWH [25]. Moreover, the distinct definitions for the VTE outcomes used in the different RCTs could explain the divergent results among the RCTs, mainly in non-critically ill patients [25,26,27]. Due to these limitations and those previously mentioned, which generate uncertainty regarding the possible benefit of therapeutic anticoagulant doses for hospitalized patients in conventional wards, further studies are needed to state more solid recommendations [25,26,27].

In addition to these limitations, the risk of bleeding should be assessed on an individualized and continuous basis, especially if higher rather than standard thromboprophylaxis doses are administered, since they are associated with a higher incidence of bleeding [41,42]. A meta-analysis involving 7781 patients hospitalized for COVID-19 showed that standard doses of thromboprophylaxis were not associated with an excess of bleeding compared to no thromboprophylaxis, while the use of therapeutic anticoagulation was associated with more than two-fold increased risk of bleeding than standard thromboprophylaxis [41]. This excess of bleeding events needs to be weighed against any potential benefit of therapeutic anticoagulant doses as a part of COVID-19 treatment. Moreover, a recent study reported that patients developing VTE after COVID-19 infection are at a higher risk for major bleeding than VTE recurrences during the first 3 months of therapy [43]. Since patients with COVID-19 infection are at an increased risk for both VTE and bleeding, the management of VTE should be always tailored to their clinical and biological characteristics. For hospitalized patients for COVID-19 with a high bleeding risk, mechanical prophylaxis should be instituted and continued until standard-doses of pharmacological thromboprophylaxis can be initiated. For hemodynamically stable COVID-19 patients with an acute VTE event and contraindication for anticoagulant treatment, an inferior vena cava filter should be placed to prevent PE. High-risk PE patients with COVID-19 and high bleeding risk require immediate reperfusion with percutaneous techniques or surgical embolectomy. In all these scenarios, clinical decisions should be based on an individual assessment of the thrombosis and bleeding risks, according to international guidelines for VTE management [7,8].

This study has some limitations. First, the selection of experts was arbitrary [11]. However, they are specialists with extensive experience in the field of VTE in different settings (primary care, hospitalization, and ICU). Second, the limitations of the DELPHI methodology have been described previously [13]. Despite this, the consensus criteria were defined a priori and the majority agreement on the seven PICO questions supports the consistency of the recommendations and suggestions. Finally, the scientific evidence in this field is evolving rapidly, so current recommendations may be modified according to the results of new RCTs.

In conclusion, the combination of PICO questions and DELPHI methodology provides a consensus on relevant and controversial issues about the use of anticoagulation in patients with COVID-19.

## Figures and Tables

**Table 1:** Domains to be addressed with their respective PICO questions.

Domain	PICO Question
**Domain 1**: thromboprophylaxis in COVID-19 patients not requiring hospital admission.	**PICO 1: Which patients with COVID-19 not requiring hospital admission should receive standard-doses of pharmacological thromboprophylaxis?** P: COVID-19 patient without hospital admission criteria I: standard-doses of pharmacological thromboprophylaxis C: recommend mobilization (avoid sedentarism) but without pharmacological thromboprophylaxis O: symptomatic VTE
**Domain 2**: thromboprophylaxis in hospitalized COVID-19 patients.	**PICO 2: In hospitalized patients for COVID-19 who were receiving anticoagulation with oral anticoagulants prior to admission, should we substitute them with LMWH?** P: COVID-19 patients admitted in a conventional ward who received previous oral anticoagulant treatment I: LMWH at anticoagulant dose C: the same oral anticoagulant treatment prior to admission O: the same goal of oral anticoagulation (prevent arterial or venous thrombosis)
	**PICO 3: What is the optimal dose of LMWH for most COVID-19 patients admitted to a conventional hospital ward?** P: COVID-19 patients admitted to a conventional hospital ward I: intermediate or therapeutic doses of LMWH C: standard doses of pharmacological thromboprophylaxis O: symptomatic VTE/need for NIMV-IOT-ECMO/mortality/major bleeding
	**PICO 4: What is the optimal dose of LMWH for most COVID-19 patients admitted to an Intensive Care Unit (ICU)?** P: COVID-19 patients admitted to an ICU I: intermediate or therapeutic doses of LMWH C: standard-doses of pharmacological thromboprophylaxis O: symptomatic VTE/need for NIMV-IOT-ECMO/mortality/major bleeding
**Domain 3**: diagnosis and treatment guide of vte in COVID-19 hospitalized patients.	**PICO 5: Which is the significance of high D-dimer levels in hospitalized patients for COVID-19?** P: patients admitted for COVID-19 I: does the D-dimer have other utilities (diagnostic or therapeutic) in this context? C: continue using D-dimer in the context of low clinical suspicion due to its high negative predictive value O: symptomatic VTE/COVID-19 severity/mortality
**Domain 4**: follow-up after discharge of patients admitted for COVID-19 with and without a vte event.	**PICO 6. Which patients without COVID-19-associated VTE should receive extended standard-doses thromboprophylaxis after hospital discharge?** P: patients without VTE during hospitalization for COVID-19 at hospital discharge I: extended thromboprophylaxis after discharge C: thromboprophylaxis during hospitalization O: symptomatic VTE/major bleeding/mortality
	**PICO 7. Which is the optimal duration of anticoagulant treatment for patients with COVID-19 associated VTE?** P: patient suffering VTE (proximal DVT and/or PE) associated with COVID-19 I: extended anticoagulant treatment (beyond the first 3 months) C: anticoagulant treatment for 3 months O: symptomatic recurrent VTE/major bleeding/clinically relevant non-major bleeding/mortality

Abbreviations. COVID-19: coronavirus disease 2019; PICO: Patient, Intervention, Comparison, Outcomes; VTE: venous thromboembolic disease; LMWH: low molecular weight heparins; NIMV-IOT-ECMO: non-invasive mechanical ventilation-orotracheal intubation-extracorporeal membrane oxygenation; ICU: Intensive Care Units.

**Table 2:** Risk factors for bleeding with anticoagulant therapy.

Advanced age (particularly >75 years)
Previous bleeding (if not associated with a reversible or treatable cause)
Recent surgery
Active cancer
Anaemia
Thrombocytopenia (particularly <50,000/µL)
Previous stroke, either haemorrhagic or ischaemic
Renal failure
Liver failure
Concomitant antiplatelet therapy or non-steroidal anti-inflammatory drugs

**Table 3:** Risk factors for venous thromboembolic disease.

Obesity
Family or personal history of VTE
Active cancer
Pregnancy/Puerperium/Estrogen replacement therapies
Recent history (<1 month) of: Major surgery Immobilization (≥3 days) due to acute medical illness
Hereditary thrombophilia (factor V Leiden, prothrombin G20210A mutation, protein C, S or antithrombin III deficiencies) or acquired (antiphospholipid syndrome).
